# Self-reported non-receipt of HIV test results: A silent barrier to HIV epidemic control in Mozambique

**DOI:** 10.1371/journal.pone.0224102

**Published:** 2019-10-22

**Authors:** Didier Mugabe, Nilesh Bhatt, James G. Carlucci, Eduardo S. Gudo, Wu Gong, Mohsin Sidat, Troy D. Moon

**Affiliations:** 1 National Institute of Health of Mozambique, Marracuene, Maputo, Mozambique; 2 Vanderbilt Institute for Global Health, Vanderbilt University Medical Center, Nashville, Tennessee, United States of America; 3 Department of Pediatrics, Division of Pediatric Infectious Diseases, Vanderbilt University Medical Center, Nashville, Tennessee, United States of America; 4 Department of Biostatistics, Vanderbilt University Medical Center, Nashville, Tennessee, United States of America; 5 Faculty of Medicine, Eduardo Mondlane University, Maputo, Mozambique; Yeshiva University Albert Einstein College of Medicine, UNITED STATES

## Abstract

**Introduction:**

People living with HIV (PLHIV) and who are aware of their HIV status can access and benefit from antiretroviral therapy (ART) with subsequent individual and public health benefits; however, many PLHIV are unaware of their HIV status. We assessed the magnitude and determinants of self-reported non-receipt of HIV test results in adults aged 15–59 years old in Mozambique.

**Methods:**

We performed a secondary analysis of data from the 2015 Mozambique Immunization indicators, Malaria and HIV/AIDS (IMASIDA 2015). Eligible adults (15–59 years) from each selected household were interviewed and data on sociodemographic characteristics, HIV knowledge, attitudes and behaviors, and HIV testing history were collected. Multivariable logistic regression assessed factors associated with self-reported non-receipt of HIV test results. Population representative estimates were calculated.

**Results:**

13,028 (90.8%) of 14,343 eligible participants were interviewed. A total of 6,654 (51.1%) respondents had previously been tested for HIV and were included in the analysis. Of these, 308 (4.6%; 95% CI: 3.70–5.77) self-reported not having received HIV test results. In the multivariable analysis, previous sexually transmitted infection (aOR: 2.76; 95% CI: 1.44–5.31), HIV stigmatizing attitudes (aOR: 1.96; 95% CI: 1.14–3.37), and lack of decision-making power towards health care seeking (aOR: 2.51; 95% CI: 1.39–4.52) were associated with non-receipt of HIV test results. Whereas, secondary or higher education (aOR: 0.25; 95% CI: 0.12–0.54), higher HIV knowledge (aOR: 0.47; 95% CI: 0.26–0.86), and age between 30–34 and 35–39 years old (aOR 0.47; 95% CI: 0.28–0.80; and aOR: 0.49; 95% CI: 0.27–0.90, respectively), were associated with receipt of HIV test results.

**Conclusions:**

In this nationally representative survey, self-reported non-receipt of HIV test results was high and of public health relevance. These findings suggest adaptation of HIV counseling and testing programs emphasizing individualized approaches that target the youngest, least educated and the poorest individuals, especially those living in rural areas.

## Introduction

Mozambique is a country of approximately 29 million people as of 2017, and has one of the highest prevalence’s of HIV in the region (13.2% in 2015) [1,2]. With support from programs such as the President’s Emergency Plan for AIDS Relief (PEPFAR) and the Global Fund to Fight AIDS, Tuberculosis, and Malaria, the Mozambican Ministry of Health (MoH) has made significant progress in the fight against HIV/AIDS. For instance, the number of people on antiretroviral therapy (ART) increased approximately four-fold, from 309,000 in 2012 to 1.2 million in 2017 [3,4], and the annual number of HIV tests performed increased from 4.6 to 6.5 million between 2016 and 2017 [5,6].

Despite this progress, there is still much work to be done to reach the UNAIDS 90-90-90 targets by 2020 (90% of people living with HIV [PLHIV) aware of their HIV status, 90% of those diagnosed with HIV receiving ART, and 90% of those on ART having undetectable viral loads). As of the end of 2017, only 47% of Mozambican adults 15–49 years old knew their HIV status, ART coverage remained relatively low with only 54% of all estimated PLHIV receiving ART, and only 66% of persons on ART were estimated to have undetectable viral loads [2,6].

HIV counseling and testing (HCT), an essential entry-point to HIV care and treatment, provides an opportunity for PLHIV to access ART in a timely manner, which has substantial benefits both at the individual and community level [7–9]. Furthermore, interventions targeting PLHIV, including counseling provided through HTC, can help one understand the meaning of their HIV status, additional risk exposure, and encourage adoption of healthier and safer behaviors. In fact, several interventions designed for PLHIV, including HTC, have been found to reduce HIV risk behaviors among HIV-positive individuals [10–11].

Although determinants of testing for HIV have been previously explored [12–15], little is known about the factors contributing to non-receipt of HIV test results despite individual acknowledgment that testing had previously been performed[16–17]. The fact that some PLHIV in high HIV prevalence regions might not be aware of their HIV status, due to non-receipt of their HIV test results, is quite alarming [18–21]. Situations like this not only represent missed opportunities for HIV diagnosis, but also puts individuals at risk for avoidable HIV related morbidity and mortality and potentially puts the community at risk of ongoing HIV transmission.

In this study, we sought to identify the magnitude and determinants of non-receipt of HIV test results after HIV testing, in adults aged 15 to 59 years old in Mozambique. To the best of our knowledge, this is the first analysis to assess determinants of self-reported not receiving HIV test results in a country with a high burden of HIV. Results from this analysis may be of importance for Mozambique and other countries in this region as they strive to improve the effectiveness of HIV testing services toward attainment of the “first 90” target by 2020.

## Methods

### Study design and sampling

We assessed the Demographic and Health Survey (DHS) database and extracted variables of interest from the 2015 Mozambique Immunization indicators, Malaria and HIV/AIDS Survey (IMASIDA 2015) [2]. IMASIDA was a nationally representative, cross-sectional, household survey of adults aged 15 to 59 years that was conducted from June 8 to September 20, 2015. A multistage cluster sampling design was used to provide representative national and provincial-level estimates, as well as stratification for rural and urban areas within provinces.

The sample size utilized for IMASIDA was based on the 2007 Mozambique General Population and Housing Census (MGPHC). A random sample of 1,640 primary sampling units (PSUs) were drawn from the MGPHC frame to form a Master Sample (MS). Each PSU in the MS consisted of 3–5 enumeration areas (EAs). On average, each EA consists of approximately 103 households. Urban EAs are larger with an average of 123 households per EA, while rural EAs are smaller with an average of 96 households. Administratively, Mozambique is divided into 11 provinces (the national capital city of Maputo has provincial status). Each province is subdivided into districts, the districts into “posto administrativos” (administrative posts), administrative posts into “localidades” (localities) or neighborhoods, and these into households.

A three-stage selection process was used based on the MS. In the first stage, 307 PSUs were randomly selected from the MS. Since the MS PSUs are large in size, one EA was selected (based on probability proportional to EA size) from each PSU to act as a sample cluster, in the second stage. This process resulted in the selection of 307 EAs, of which 134 EAs were from urban areas and 173 EAs were from rural areas. Samples were selected independently from EAs in every stratum. In the third stage, a fixed number of 24 households per EA were selected with equal probability from a previous systematic household listing process, where all households in each of the 307 selected EAs were listed. This process resulted in a selection of 7,368 households. Only pre-selected households were approached. No replacements or changes of the preselected households were allowed in the implementing stages in order to prevent selection bias. Details of the sampling design are described elsewhere [2].

All adult men and women aged 15–59 years who were permanent residents, or those who spent the night prior to the survey in the household were eligible. All eligible participants were informed about the survey objectives and procedures, and were asked to voluntary participate. A household questionnaire plus an individual female and/or male questionnaire were administered to consented participants to collect data on: socioeconomic and demographic characteristics; HIV knowledge, attitudes and behaviors; participant sexual behaviors and sexually transmitted infections (STIs); as well as stigmatizing attitudes towards PLHIV.

### HIV testing in Mozambique

In Mozambique, HIV testing is offered 1) at Voluntary Counseling and Testing Units at health facilities; 2) through community-based voluntary counseling and testing; and 3) at all clinical care offices at the health facilities, through provider-initiated HIV testing. The primary mode of HIV testing in adults is though rapid diagnostic tests (RDT) utilizing the Determine^™^ and Uni-Gold^™^ test kits in accordance with national HIV testing guidelines. According to these guidelines, the HIV testing process is divided into three phases. In the first phase (pre-counseling), consent for HIV testing is obtained and the patient is offered information about the testing process, possible results and how to cope with each potential result. In the second phase (testing), the HIV test is performed in front of the patient and additional information on how to interpret the HIV results is provided to the patient. In the third phase (post-test), the patient is given the test results and depending on the outcome, the health care worker provides information about linkage to HIV care and treatment services, if needed, or counseling on best practices for HIV prevention.

### Measurements

The primary outcome of interest was participant self-report of not receiving an HIV test result. This was defined as individuals aged 15–59 years old who reported that they had previously been tested for HIV but did not receive their HIV test result. This was assessed through a series of questions which included “have you ever been tested for HIV?” and “did you receive your HIV test results?”. For those participants who respondeded “Yes” to the first question and “No” to the second question, they were classified as cases, and were included in this secondary analysis. Respondents who did not meet this criteria were excluded.

Covariates included socio-demographic factors such as gender, age, educational level, marital status, province of residence, and urban versus rural residence. Additional covariates included HIV knowledge: assessing respondent’s knowledge about prevention and modes of HIV transmission (see Appendix A); individual HIV related stigma: assessing respondent’s stigma and discrimination attitudes towards PLHIV (see Appendix B); media exposure: evaluating respondent’s frequency of reading a newspaper, listening to radio and/or watching TV (see Appendix C); and HIV disclosure and confidentiality concerns (see Appendix D). Respondents’ HIV status, based on testing occurred during the IMASIDA survey, was also included.

### Statistical analyses

Statistical analyses were performed using STATA software version 15.1 (Stata Corporation, College Station, Texas, USA). All analyses accounted for the survey sampling scheme consisting of multistage stratification, clustering, and weighting. We used subpopulation analysis to calculate estimates for subsets of those ever tested for HIV, those who received, and those who did not receive HIV test results. Although only cases in each subset were included in the calculation of the respective estimates, all cases in the dataset were used in the calculation of standard errors for the estimates in each subset. This ensures representativeness of our estimates to the population of Mozambicans aged 15 and 59 years.

Frequencies and percentages were used to describe participants’ sociodemographic characteristics as well as HIV risk behaviors. All variables were categorized, and weighted percentages were reported based on the inverse of the selection probability. Comparisons between individuals who reported not receiving an HIV test result and those who reported having received HIV test results were performed using Pearson’s chi-square test, with p-values reported.

Logistic regression analysis, with robust variance estimation to account for the survey sampling scheme, was used to identify factors associated with self-report of not receiving HIV test results. Potential explanatory variables selected *a priori* were included in the bivariate analysis. Two criteria were used for including variables in the multivariate model: a p-value less than 0.1 in the bivariate analysis and noncollinearity, defined as a Pearson's R correlation coefficient (R) less than 0.5. If two variables were found correlated, with R greater than 0.5, the variable strongly associated with the outcome (smaller p-value) was retained. Based on the correlation coefficient criteria, wealth index and marital status were not included in the multivariate regression model due to high correlation and/or interaction with some variables. Age, sex and HIV status were included in the multivariate model based on their relevance as potential predictors, even though they were not statistically significant in the bivariate analysis (p-value >0.1). The multivariate model included gender, age, educational level, province of residence, urban or rural, primary language, occupation, number of sexual partners, condom use, history of STI, HIV status, stigma, confidentiality, media exposure, HIV knowledge, decision to seek health care, and accounted for adjustment for confounders. The odds ratios (OR) with two-sided 95% confidence intervals (CI) were reported. A p-value less than 0.05 was considered statistically significant. Both 95% CIs and the p-values were calculated accounting for the study design and subpopulation analysis.

### Ethics approval

Ethical approval to conduct the 2015 IMASIDA survey was obtained from the National Committee for Bioethics in Health of Mozambique (*Comité Nacional de Bioética para Saúde*, *CNBS*) (Application number: 127/CNBS/2013), the Institutional Review Board (IRB) of ICF International, and the US Centers for Disease Control and Prevention (CDC). For this secondary data analysis, the database was de-identified and no informed consent was required from participants. Additional ethics review for this study was provided by the Vanderbilt University Institutional Review Board (IRB: 190017).

## Results

**Fig 1:**
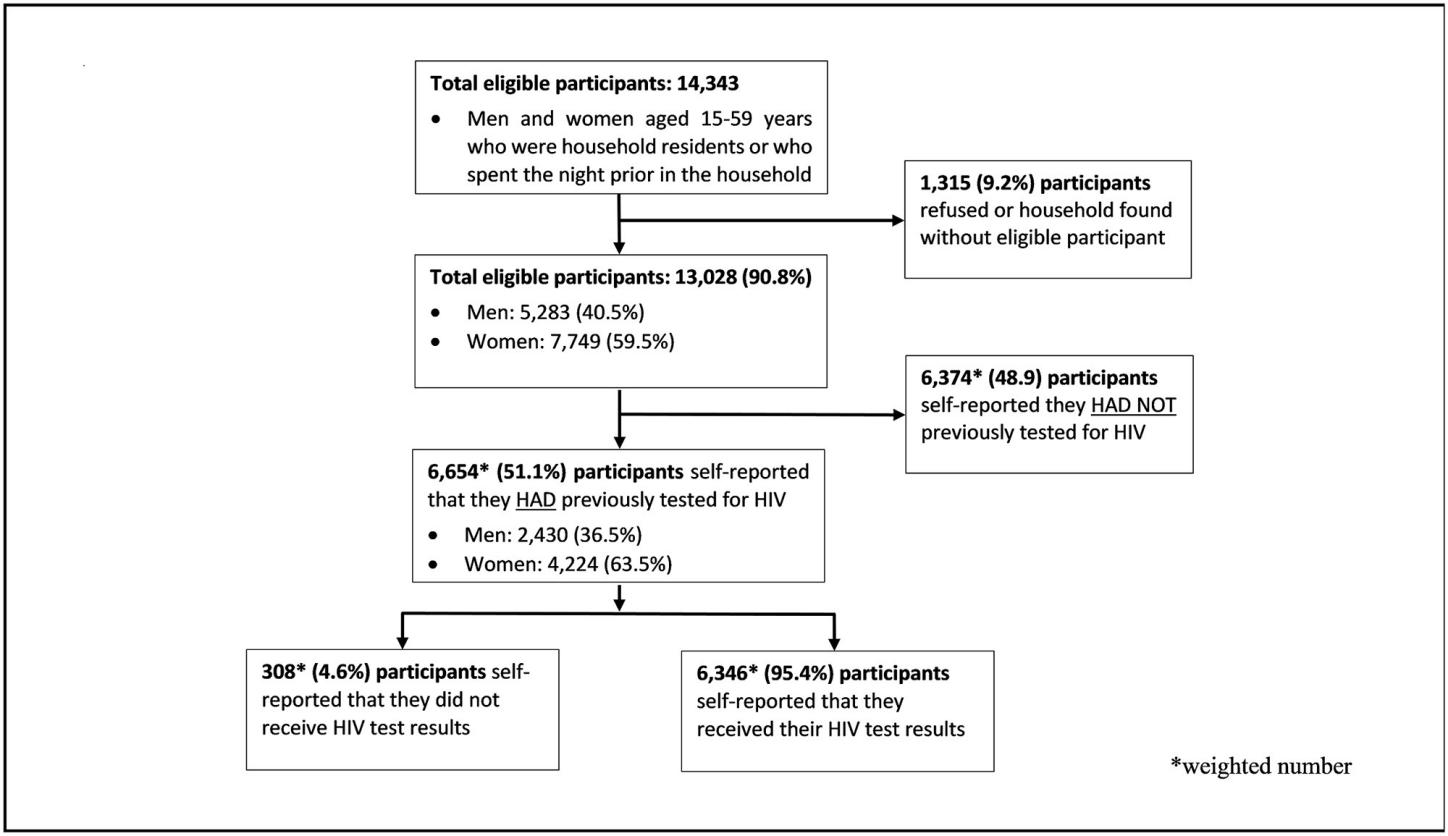
Flow chart for eligibility and enrolment for self-reported non-recipients of HIV test results.

Of the 7,368 households originally selected, a total of 7,342 (99.6%) were occupied at the time of fieldwork and 7,169 (97.6%) were successfully interviewed. Within these households, 14,343 persons were identified as eligible for individual interviews, of whom 13,028 (90.8%) completed the interview (5,283 men and 7,749 women). A total of 6,654 (51.1%) participants reported having been tested for HIV at some point prior to the survey and were included in our analysis. Of these, 6,346 (95.4%, 95% CI: 94.2–96.3) reported having received their HIV test results, and 308 (4.6%; 95% CI: 3.7–5.8) reported not having received their HIV test results (Fig 1).

Among those previously tested for HIV, 4,224 (63.5%) were female, 2,171 (32.6%) were between 15–24 years old, 4,355 (65.4%) had no more than primary education, and 3,652 (54.9%) lived in rural areas. Education level, marital status, residence, primariry language, occupation and wealth index varied among respondents who self-reported non-receipt and those who received HIV test result (p<0.001). The majority of respondents who self-reported non-receipt of HIV test results were: less educated (26.6% with primary and 26% with no education), married or living with a partner (75.7%), living in rural areas (74.7%), primarily spoke a local language (94.8%), were unemployed (42.2%), and the majority were from lower wealth indexs [19.6% (poorest), 26.5% (poor) and 21.7% (midle)] (Table 1).

**Table 1:** Respondents’ social and demographic characteristics.

Characteristics	All cases	Received HIV test results		p-value
	(n = 6,654)	No (n = 308)	Yes (n = 6,346)	
	N (%)	N (%)	N (%)	
**Sex**				0.221
Male	2430 (36.5)	99 (32.1)	2331 (36.7)	
Female	4224 (63.5)	209 (67.9)	4015 (63.3)	
**Age group (in years)**				0.251
15–24	2171 (32.6)	116 (37.5)	2056 (32.4)	
25–29	1149 (17.3)	44 (14.2)	1106 (17.4)	
30–34	929 (14.0)	32 (10.4)	897 (14.1)	
35–39	834 (12.5)	40 (13.1)	794 (12.5)	
40+	1571 (23.6)	77 (24.9)	1494 (23.5)	
**Education**				<0.001
No formal education	1097 (16.5)	82 (26.6)	1015 (16.0)	
Primary	3258 (49.0)	181 (58.9)	3077 (48.5)	
Secondary or higher	2299 (34.6)	45 (14.5)	2254 (35.5)	
**Marital status**				0.044
Never married	1052 (15.8)	33 (10.6)	1019 (16.1)	
Currently married	4623 (69.5)	233 (75.7)	4390 (69.2)	
Formerly married	979 (14.7)	42 (13.7)	937 (14.8)	
**Residence**				<0.001
Urban	3002 (45.1)	78 (25.3)	2924 (46.1)	
Rural	3652 (54.9)	230 (74.7)	3422 (53.9)	
**Primary language**				<0.001
Portuguese	941 (14.1)	16 (5.2)	925 (14.6)	
Other	5698 (85.6)	292 (94.8)	5406 (85.2)	
Missing	15 (0.2)	0 (0.0)	15 (0.2)	
**Employed in the last 12 months**				0.299
No	2491 (37.4)	130 (42.2)	2361 (37.2)	
Yes	4163 (62.6)	178 (57.8)	3985 (62.8)	
**Occupation**				0.006
Unemployed	2491 (37.4)	130 (42.2)	2361 (37.2)	
Professional	423.4 (6.4)	12 (3.7)	412 (6.5)	
Sales	1144 (17.2)	38 (12.2)	1106 (17.4)	
Agriculture	1257 (18.9)	86 (27.9)	1171 (18.5)	
Manual work	1322 (19.9)	43 (13.9)	1279 (20.2)	
Missing	17 (0.3)	0 (0.0)	17 (0.3)	
**Wealth index**				<0.001
Poorest	854 (12.8)	60 (19.6)	793 (12.5)	
Poor	962 (14.5)	82 (26.5)	880 (13.9)	
Middle	1123 (16.9)	67 (21.7)	1056 (16.7)	
Richer	1539 (23.1)	46 (15.0)	1493 (23.5)	
Richest	2176 (32.7)	53 (17.3)	2123 (33.5)	

Moreover, HIV stigmatizing atitudes, confidentiality concern, HIV knowledge and media exposure were associated with self report non-receipt of HIV test results (p<0.007). While the majority of respondents who received their HIV test results had the lowest level of HIV stigmatizing attitudes [44.1% (low) and 48.9% (medium)], HIV disclosure concern was more common (79.4%) among those who did not receive HIV results (Table 2). Respondents reporting non-receipt of their HIV test result more frequently had no exposure to the media (56.4%) and had lower HIV knowledge (42.8%) (Table 2). No differences in respondents’ current HIV status (based on 2015 IMASIDA HIV testing and not on prior reported HIV testing) were observed among groups (p = 0.868) (Table 2).

**Table 2:** Respondents’ HIV knowledge, attitudes and sexual behaviors.

Characteristics	All cases	Received HIV test results		p-value
	(n = 6,654)	No (n = 308)	Yes (n = 6,346)	
	N (%)	N (%)	N (%)	
**Number of sexual partners including spouse, in the last 12 months**				0.088
None	893 (13.4)	48 (15.6)	845 (13.3)	
One	4998 (75.1)	238 (77.3)	4760 (75.0)	
Two or more	757 (11.4)	22 (7.1)	735 (11.6)	
Missing	6 (0.1)	0 (0.0)	6 (0.1)	
**Number of sexual partners excluding spouse, in last 12 months**				0.048
None	4900 (73.6)	249 (80.7)	4651 (73.3)	
One	1451 (21.8)	49 (16.0)	1402 (22.1)	
Two or more	297 (4.5)	10 (3.2)	287 (4.5)	
Missing	6 (0.1)	0 (0.0)	6 (0.1)	
**Used condom in the last sex with most recent partner**				0.005
Yes	1111 (16.7)	29 (9.5)	1081 (17.0)	
No	4648 (69.9)	231 (75.0)	4417 (69.6)	
Missing	895 (13.5)	48 (15.6)	848 (13.4)	
**Had STI in the last 12 months**				0.053
Yes	300 (4.5)	21 (6.8)	279 (4.4)	
No	6317 (94.9)	284 (92.3)	6033 (95.1)	
Missing	37 (0.6)	3 (1.0)	34 (0.5)	
**Time from the last HIV test**				0.019
Less than 12 months	3838 (57.7)	150 (48.7)	3688 (58.1)	
More than 12 moths	2816 (42.3)	158 (51.3)	2658 (41.9)	
**HIV status**				0.868
Positive	1010 (15.2)	51 (16.6)	959 (15.1)	
Negative	4785 (71.9)	234 (76.0)	4551 (71.7)	
Missing	859 (12.9)	23 (7.5)	836 (13.2)	
**HIV stigmatizing attitudes**				<0.001
Low	2870 (43.1)	71 (22.9)	2799 (44.1)	
Medium	3316 (49.8)	211 (68.6)	3105 (48.9)	
High	406 (6.1)	17 (5.4)	389 (6.1)	
Missing	63 (0.9)	10 (3.2)	53 (0.8)	
**HIV disclosure/confidentiality concern**				0.007
No	1509 (22.7)	48 (15.5)	1461 (23.0)	
Yes	4858 (73.0)	245 (79.4)	4614 (72.7)	
Missing	287 (4.3)	16 (5.2)	271 (4.2)	
**HIV knowledge/awareness**				<0.001
Low	1477 (22.2)	132 (42.8)	1345 (21.2)	
Medium	2697 (40.5)	103 (33.3)	2595 (40.9)	
High	2387 (35.9)	64 (20.7)	2324 (36.6)	
Missing	93 (1.4)	10 (3.25)	82 (1.29)	
**Media exposure**				<0.001
No exposure	2196 (33.0)	174 (56.4)	2022 (31.9)	
Medium	2694 (40.5)	98 (31.9)	2596 (40.9)	
High	1750 (26.3)	36 (11.7)	1714 (27.0)	
Missing	14 (0.2)	0 (0.0)	14 (0.2)	
**Decision to seek health care**				0.001
Respondent alone	1060 (15.9)	33 (10.7)	1027 (16.2)	
Respondent and another person	2845 (42.8)	135 (43.8)	2710 (42.7)	
Other person	708 (10.6)	65 (21.2)	643 (10.1)	
Missing	2041 (30.7)	75 (24.4)	1966 (31.0)	

In the multivariable analysis, having HIV stigmatizing attitudes (aOR: 1.96; 95% CI: 1.14–3.37), previous STI (aOR: 2.76; 95% CI: 1.44–5.31), and not having decision-making power with regards to health care seeking (aOR: 2.51; 95% CI: 1.39–4.52) were associated with self-reported non-receipt of HIV test results. On the other hand, respondents’ age between 30–34 and 35–39 years old (aOR 0.47; 95% CI: 0.28–0.80; and aOR: 0.49; 95% CI: 0.27–0.90, respectively), secondary or higher education (aOR: 0.25; 95% CI: 0.12–0.54), and high HIV knowledge (aOR: 0.47; 95% CI: 0.26–0.86) were associated with receiving HIV test results. Residing in Manica province was also associated with receiving HIV test results, when compared to residing in the capital, Maputo City (aOR: 0.23; 95% CI 0.06–0.87) (Table 3).

**Table 3:** Determinants of self-reported non-receipt of HIV test results among individuals aged 15–59 years old: Bivariable (unadjusted) and multivariable (adjusted) logistic regression analysis.

Characteristics	Unadjusted		Adjusted	
	OR (95% CI)	*P*-Value	aOR (95% CI)	*P*-Value
**Sex**				
Female	ref		ref	
Male	0.82 (0.59–1.13)	0.222	1.56 [0.94–2.59)	0.086
**Age group (in years)**				
15–24	ref		ref	
25–29	0.70 (0.47–1.06)	0.090	0.66 [0.39–1.11)	0.116
30–34	0.64 (0.40–1.02)	0.061	0.47 (0.28–0.80)	0.006
35–39	0.90 (0.59–1.38)	0.639	0.49 (0.27–0.90)	0.020
40+	0.91 (0.65–1.28)	0.594	0.66 (0.39–1.10)	0.112
**Education**				
No Formal education	ref		ref	
Primary	0.73 (0.53–1.01)	0.054	0.93 (0.57–1.51)	0.757
Secondary or higher	0.25 (0.16–0.39)	<0.001	0.25 (0.12–0.54)	<0.001
**Marital status**				
Never married	ref			
Currently married	1.66 (1.15–2.39)	0.007		
Formerly married	1.41 (0.88–2.24)	0.149		
**Province**				
Maputo City	ref		ref	
Niassa	3.10 (1.80–5.34)	<0.001	0.98 (0.32–2.97)	0.968
Cabo Delgado	5.33 (2.60–10.91)	<0.001	1.02 (0.30–3.41)	0.975
Nampula	3.02 (1.35–6.78)	0.008	0.69 (0.22–2.18)	0.523
Zambézia	1.85 (0.94–3.61)	0.073	0.46 (0.14–1.54)	0.205
Tete	2.55 (1.25–5.19)	0.010	0.79 (0.24–2.62)	0.700
Manica	0.80 (0.39–1.66)	0.553	0.23 (0.06–0.87)	0.030
Sofala	5.19 (1.96–13.75)	0.001	1.39 (0.38–5.07)	0.620
Inhambane	1.69 (0.83–3.47)	0.150	0.44 (0.13–1.48)	0.185
Gaza	1.84 (0.99–3.40)	0.052	0.53 (0.15–1.92)	0.333
Maputo Province	2.19 (1.26–3.82)	0.006	1.34 (0.47–3.80)	0.578
**Residence**				
Urban	ref		ref	
Rural	2.52 (1.67–3.81)	<0.001	1.69 (0.96–2.98)	0.068
**Primary language**				
Portuguese	ref		ref	
Other	3.23 (1.88–5.53)	<0.001	1.31 (0.49–3.48)	0.587
**Wealth index**				
Poorest	ref			
Poor	1.21 (0.81–1.82)	0.345		
Middle	0.83 (0.42–1.65)	0.593		
Richer	0.40 (0.24–0.69)	0.001		
Richest	0.33 (0.20–0.55)	<0.001		
**Decision to seek health care**				
Respondent alone	ref		ref	
Respondent and another person	1.55 (0.89–2.68)	0.120	1.84 (0.94–3.63)	0.077
Other person	3.16 (1.83–5.45)	<0.001	2.51 (1.39–4.52)	0.002
**Lifetime sexual partners**				
None				
One	0.96 (0.43–2.15)	0.918		
Two or more	0.74 (0.29–1.89)	0.533		
**Number of sexual partners including spouse, in the last 12 months**				
None	ref			
One	0.88 (0.60–1.28)	0.509		
Two or more	0.53 (0.30–0.91)	0.021		
**Number of sexual partners excluding spouse, in last 12 months**				
None	ref		ref	
One	0.66 (0.47–0.93)	0.018	0.84 (0.40–1.77)	0.648
Two or more	0.63 (0.29–1.36)	0.238	1.03 (0.13–8.27)	0.976
**Used condom in the last sex with most recent partner**				
No	ref		ref	
Yes	0.52 (0.32–0.82)	0.006	0.89 (0.41–1.91)	0.763
**Had STI in the last 1 month**				
No	ref		ref	
Yes	1.59 (0.99–2.55)	0.055	2.76 (1.44–5.31)	0.002
**Time from the last HIV test**				
Less than 12 months	ref		ref	
More than 12 moths	1.46 (1.06–2.01)	0.019	1.49 (0.97–2.29)	0.070
**HIV status**				
Negative	ref		ref	
Positive	1.03 (0.71–1.49)	0.868	1.30 (0.79–2.14)	0.307
**Employment in the last 12 months**				
No	ref			
Yes	0.81 (0.55–1.20)	0.299		
**Occupation**				
Unemployed	ref		ref	
Professional	0.51 (0.27–0.95)	0.033	0.83 (0.20–3.52)	0.804
Sales	0.62 (0.37–1.03)	0.066	0.74 (0.39–1.40)	0.354
Agriculture	1.34 (0.81–2.19)	0.251	1.14 (0.57–2.28)	0.709
Manual work	0.61 (0.39–0.95)	0.031	0.61 (0.31–1.23)	0.171
**HIV stigmatizing attitudes**				
Low	ref		ref	
Medium	2.70 (1.81–4.01)	<0.001	1.96 (1.14–3.37)	0.016
High	1.68 (0.91–3.11)	0.098	0.55 (0.16–1.91)	0.343
**HIV disclosure/confidentiality concern**				
No	ref		ref	
Yes	1.62 (1.14–2.31)	0.008	1.12 (0.67–1.85)	0.667
**Media exposure**				
No exposure	ref		ref	
Low exposure	0.44 (0.31–0.62)	<0.001	0.70 (0.44–1.13)	0.146
High	0.24 (0.16–0.38)	<0.001	0.85 (0.46–1.57)	0.594
**HIV knowledge/awareness**				
Low	ref		ref	
Medium	0.40 (0.28–0.58)	<0.001	0.64 (0.40–1.02)	0.061
High	0.28 (0.18–0.44)	<0.001	0.47 (0.26–0.86)	0.014

All estimates were calculated accounting for the survey sampling scheme

Futhermore, residing in a rural area (OR: 1.69; 95% CI: 0.96–2.98), primarily speaking a local language (OR: 1.31; 95% CI: 0.49–3.48), being a farmer (OR: 1.14; 95% CI: 0.57–2.28), and having concern about HIV disclosure (OR: 1.12; 95% CI: 0.67–1.85) trended toward being associated with not receiving HIV test results. Similarly, high media exposure (OR: 0.85; 95% CI: 0.46–1.57) trended toward an association with receiving HIV test results (Table 3).

## Discussion

In this large, nationally representative survey of Mozambican adults, 4.6% of respondents who self-reported that they had previously been tested for HIV, also reported that they did not receive the results of that HIV test. This phenomenon is both perplexing and troublesome for a variety of reasons. First, in Mozambique, the primary modality for HIV testing is a rapid diagnostic test (RDT) that provides test results within minutes, eliminating the need to return for one’s results. Second, while 4.6% may appear to be a small proportion, when scaled to Mozambique’s 2015 population of 13 million adults aged 15–59 years [22], and assuming that 51% (6.6 million) of these had previously tested for HIV (findings from this study), then 4.6% represents approximately 307,000 individuals who were tested for HIV but did not receive their HIV test results. Furthermore, considering an HIV prevalence of 16.6% (95% CI: 15.99–18.96) among individuals who did not receive their HIV test results (findings from this study), then approximately 51,000 HIV-positive persons in Mozambique were tested but did not receive their HIV test results, and therefore do not know their HIV status. This represents a missed opportunity to diagnose and treat a sizeable group of PLHIV that have already accessed the health care system. If Mozambique is to achieve real and sustained progress towards the first 90 of the UNAIDS 90-90-90 targets, then this observed diagnostic gap must be addressed [23–24].

While the reasons for non-receipt of HIV test results cannot be definitively determined through this study, we found that individual-level factors such as younger age (15–24 years), lower educational attainment, lower HIV knowledge, higher stigmatizing attitudes towards PLHIV, higher sexual risk behaviors, and lower decision-making power with regards to one’s health were associated with self-reported non-receipt of HIV test results. While no previous studies have sought to identify determinates of non-receipt of HIV test results in regions with high burden of HIV, prior studies have investigated determinants of HIV testing and identified analogous individual-level factors associated with not testing for HIV [13,25–31] and with failure to return for post-test HIV counseling [32–33]. Therefore, the cumulative evidence suggests that there is a consistent pattern of individual-level barriers to seeking, receiving, and/or understanding HIV testing and HIV test results. Tailored messaging and interventions aimed at addressing these individual-level factors have the potential to improve overall HIV testing uptake and follow-through with receiving and understanding test results. We emphasize the importance of ensuring that individuals who are tested for HIV, whether by counselors in the community or by health care providers at a health facility, receive their HIV test results on the same day before leaving the testing site, and we advocate for improved assurances and documentation from the person conducting the test that the test result was delivered and understood.

Other potential factors that could be associated with non-receipt of HIV test results might include the setting where HIV testing was performed and/or the training and experience of the person performing the test and providing counseling. However, these factors were not included in our analysis due to insufficient data collected with respect to these variables in the dataset.

Another limitation of our study is that self-reported non-receipt of test results cannot be reliably distinguished from intentional non-disclosure of prior test results or unintentional non-disclosure of test results due to a lack of understanding. There is a possibility that some HIV-positive individuals who were actually aware of their prior HIV test results falsely reported that they did not receive their HIV test result, either because they did not want to share their HIV status with the interviewer or because they were hoping to be tested again to confirm the results. In support of this hypothesis, we found an increased likelihood of self-reported non-receipt of HIV test results among persons who had concerns about HIV disclosure/confidentiality. However, this explanation seems less likely since there were no differences in confirmed HIV status between those who received and those who did not receive HIV test results.

Another possible explanation for persons reporting non-receipt of HIV test results is that they did not understand the significance of HIV testing or HIV test results. We found that as HIV knowledge increased, the likelihood of reporting receipt of HIV test results was greater. Additionally, Mozambique is a country in which the official language used by health providers is Portuguese, yet many patients may only speak one of several indigenous languages. While efforts have been made over the last decade to ensure that health providers speak the indigenous languages of the areas in which they work, this is not always the case. As such, when low HIV knowledge on the part of the patient is coupled with health care offered in a language the patient may not fully understand, it is easy to see how someone could be confused as to whether or not they received their HIV test result. Further study is needed to differentiate between true non-receipt of HIV test results versus not understanding HIV test results.

To the best of our knowledge, this is the first study to specifically describe determinants of self-reported non-receipt of HIV test results, in a region with high burden of HIV. However, other countries in similar contexts including Zambia, South Africa, Tanzania, Angola, and Eswatini have reported similar rates of self-reported non-receipt of HIV test results in their national surveys [18,21–34]. Even accounting for a small portion of survey response error, the observed 4.6% burden of self-reported non-receipt of HIV test results constitutes evidence of an under-studied problem in HIV testing and counselling services that needs to be urgently addressed in order to achieve the “first 90%”, a fundamental step in achieving the UNAIDS 90-90-90 goals, and subsequent HIV epidemic control, especially in regions with high burden of HIV.

## Conclusion

In this nationally representative survey, the burden of self-reported non-receipt of HIV test results was high and of public health relevance. Adaptation of HIV counseling and testing programs emphasizing individualized approaches that target the youngest, least educated and the poorest individuals, especially those living in rural areas, and reinforcement of effective delivery and communication of HIV test results are needed.

## Supporting information

S1 Appendix HIV hnowledge scale. (DOCX)

S2 Appendix HIV stigmatizing attitude scale. (DOCX)

S3 Appendix Media exposure scale. (DOCX)

S4 Appendix HIV/AIDS disclousure and confidentiality. (DOCX)
