# Supplementary material for: Self-reported non-receipt of HIV test results: A silent barrier to HIV epidemic control in Mozambique
Source: PLoS One. 2019 Oct 22;14(10):e0224102. doi: 10.1371/journal.pone.0224102 (PMC6804976; doi:10.1371/journal.pone.0224102)
Supplement: S1 Appendix — (DOCX) [file pone.0224102.s001.docx]

**Appendix A: HIV knowledge scale**

HIV knowledge was assessed through an eight-question scale. Each item assessed the respondent’s knowledge about prevention and modes of HIV transmission. For example, “Can people get HIV by sharing food with someone who has HIV/AIDS?”, or “Can people get AIDS by witchcraft or other supernatural means?”. Each correct response was assigned one point with “don't know” responses scored as incorrect. Points were then summed to create an HIV knowledge score ranging from 0 to 8. HIV knowledge was classified into three levels: low (4 or less correct responses); medium (5 to 6 correct responses), and high (7 to 8 correct responses) (See Table 1, below). This instrument has previously been used in other studies published in peer-reviewed journals [1-3].

**Table 1. Items included in HIV knowledge scale**

| Reduce risk of getting HIV by always use condoms during sex  1 if correct  0 if wrong |
| --- |
| Reduce chances of HIV by having just one uninfected sex partner  1 if correct  0 if wrong |
| Can get HIV from mosquito bites  1 if correct  0 if wrong |
| Can get HIV by sharing food with person who has aids  1 if correct  0 if wrong |
| Can a healthy-looking person have HIV  1 if correct  0 if wrong |
| Can get HIV by witchcraft or supernatural means  1 if correct  0 if wrong |
| Circumcision protects men against HIV  1 if correct  0 if wrong |
| HIV can be transmitted from mother to baby:  a. During pregnancy  b. During delivery  c. By breastfeeding  1 if a, b and c are correct  0 if a or b or c is wrong / don’t know |
| **HIV Knowledge level**  Low = 4 or less correct responses  Medium = 5-6 correct responses  High = 7-8 correct responses |

___________________________________

1. Lépine A, Terris-Prestholt F, Vickerman P. Determinants of HIV testing among Nigerian couples: a multilevel modelling approach. Health Policy Plan. 2015;30: 579–592. doi:10.1093/heapol/czu036

2. Gazimbi MM, Magadi MA. A Multilevel Analysis of the Determinants of HIV Testing in Zimbabwe: Evidence from the Demographic and Health Surveys. HIV/AIDS Research and Treatment – Open Journal. 2017;4: 14–31. doi:10.17140/HARTOJ-4-124

3. Peltzer K, Matseke G, Mzolo T, Majaja M. Determinants of knowledge of HIV status in South Africa: results from a population-based HIV survey. BMC Public Health. 2009;9: 174. doi:10.1186/1471-2458-9-174
