# Supplementary material for: Self-reported non-receipt of HIV test results: A silent barrier to HIV epidemic control in Mozambique
Source: PLoS One. 2019 Oct 22;14(10):e0224102. doi: 10.1371/journal.pone.0224102 (PMC6804976; doi:10.1371/journal.pone.0224102)
Supplement: S2 Appendix — (DOCX) [file pone.0224102.s002.docx]

**Appendix B: HIV stigmatizing attitude scale**

Individual HIV related stigma was assessed through five items which captures stigma and discrimination attitudes towards PLHIV. For example, if a respondent “would be willing to take care of a family member with HIV?” or "Should a female teacher with HIV be allowed to continue teaching?”. Each stigmatizing response of “no” or “don’t know” was attributed one point and the “yes” response, zero. A total score was calculated by summing all the points (range 0–5) and coded into three levels of stigmatizing attitudes: 0-1 low, 2-3 medium, and 4-5 high (see Table 2, below). This instrument has previously been used in other studies published in peer-reviewed journals [1-3].

**Table 2. Items included in the HIV stigmatizing attitude scale**

| Would buy vegetables from vendor with HIV  1 if no or don’t know  0 if yes |
| --- |
| HIV-positive family member kept secret  1 if yes or don’t know  0 if no |
| Willing to care for HIV- positive relative in household  1 if no or don’t know  0 if yes |
| Female teacher with HIV allowed to continue teaching  1 if no or don’t know  0 if yes |
| HIV-positive women should breastfeed their babies  1 if no or don’t know  0 if yes |
| **HIV stigma attitude level**  Low = 0-1 points  Medium = 2-3 points  High = 4-5 points |

___________________________________

1. Lépine A, Terris-Prestholt F, Vickerman P. Determinants of HIV testing among Nigerian couples: a multilevel modelling approach. Health Policy Plan. 2015;30: 579–592. doi:10.1093/heapol/czu036

2. Gazimbi MM, Magadi MA. A Multilevel Analysis of the Determinants of HIV Testing in Zimbabwe: Evidence from the Demographic and Health Surveys. HIV/AIDS Research and Treatment – Open Journal. 2017;4: 14–31. doi:10.17140/HARTOJ-4-124

3. Peltzer K, Matseke G, Mzolo T, Majaja M. Determinants of knowledge of HIV status in South Africa: results from a population-based HIV survey. BMC Public Health. 2009;9: 174. doi:10.1186/1471-2458-9-174
