# Supplementary material for: Self-reported non-receipt of HIV test results: A silent barrier to HIV epidemic control in Mozambique
Source: PLoS One. 2019 Oct 22;14(10):e0224102. doi: 10.1371/journal.pone.0224102 (PMC6804976; doi:10.1371/journal.pone.0224102)
Supplement: S3 Appendix — (DOCX) [file pone.0224102.s003.docx]

**Appendix C: Media exposure scale**

Media exposure was evaluated based on a score created from the frequency of reading a newspaper, listening to radio and/or watching TV. An additive scale of three items relating to the frequency of watching television, reading newspapers, and listening to the radio was used (range: 0-6). Media exposure was classified into three levels: 0 = no exposure, 1-3 = some exposure, and 4-6 = high exposure (see Table 3, below). This method has previously been used in other studies published in peer-reviewed journals [1-3].

**Table 3: Items included in media exposure scale**

| Frequency of reading newspaper or magazine  Not at all - 0  Less than once a week - 1  At least once a week - 2 |
| --- |
| Frequency of listening to radio  Not at all - 0  Less than once a week - 1  At least once a week - 2 |
| Frequency of watching television  Not at all - 0  Less than once a week - 1  At least once a week - 2 |
| **Media exposure level**  No exposure = 0  Some exposure = 1-3  High exposure = 4-6 |

___________________________________

1. Lépine A, Terris-Prestholt F, Vickerman P. Determinants of HIV testing among Nigerian couples: a multilevel modelling approach. Health Policy Plan. 2015;30: 579–592. doi:10.1093/heapol/czu036

2. Gazimbi MM, Magadi MA. A Multilevel Analysis of the Determinants of HIV Testing in Zimbabwe: Evidence from the Demographic and Health Surveys. HIV/AIDS Research and Treatment – Open Journal. 2017;4: 14–31. doi:10.17140/HARTOJ-4-124

3. Peltzer K, Matseke G, Mzolo T, Majaja M. Determinants of knowledge of HIV status in South Africa: results from a population-based HIV survey. BMC Public Health. 2009;9: 174. doi:10.1186/1471-2458-9-174
