# Supplementary material for: Self-reported non-receipt of HIV test results: A silent barrier to HIV epidemic control in Mozambique
Source: PLoS One. 2019 Oct 22;14(10):e0224102. doi: 10.1371/journal.pone.0224102 (PMC6804976; doi:10.1371/journal.pone.0224102)
Supplement: S4 Appendix — (DOCX) [file pone.0224102.s004.docx]

**Appendix D: HIV/AIDS disclosure and confidentiality**

A respondent’s HIV/AIDS disclosure and confidentiality concerns were accessed through a single question. Participants were asked if they would want others to know if a family member became infected with HIV. Responses were coded as “yes” if respondent would want others to know a family member became infected and “no” if they did not.
